# THE EFFECT OF OBESITY ON COVID-19 OUTCOMES AMONG HOSPITALIZED OLDER ADULTS

**DOI:** 10.1093/geroni/igac059.2694

**Published:** 2022-12-20

**Authors:** Mary Parianos, Ali Vaeli Zadeh, Ricardo Criado Carrero, Claudia Marcano, Andrew Crawford, Elias Collado, Joshua Larned

**Affiliations:** University of Miami, Fort Lauderdale, Florida, United States; Holy Cross Health-Jim Moran Cardiovascular Research Institute, Fort Lauderdale, Florida, United States; University of Miami/Holy Cross Hospital, Fort Lauderdale, Florida, United States; University of Miami, Fort Lauderdale, Florida, United States; Holy Cross Health-University of Miami, Fort Lauderdale, Florida, United States; Holy Cross Health-Jim Moran Cardiovascular Research Institute, Fort Lauderdale, Florida, United States; Holy Cross Health-University of Miami, Fort Lauderdale, Florida, United States

## Abstract

**Background:**

According to the CDC, approximately 30% of hospitalizations for COVID-19 infection between the onset of the pandemic and November 2020 were attributed to obesity. However, there is limited data on how obesity affects the overall outcome of COVID-19 in hospitalized older adults.

**Methods:**

A retrospective study was conducted using the PearlDiver database (PearlDiver Technologies, Fort Wayne, IN). Using ICD-10 codes, a cohort of patients aged 65–75 and Elixhauser Comorbidity Index (ECI) >4 with a history of obesity admitted for COVID-19 was identified. This cohort was matched with a group of patients with no history of obesity, considering age, gender, and ECI. Records from both groups were reviewed for multiple outcomes over 30 days following admission. Pearson’s chi-squared was used to compare groups. The strength of association was reported using Risk Ratios (RR). A p-value < 0.05 was deemed significant.

**Results:**

There were 151,429 members in each group. Obese individuals had a higher risk of 30-day all-cause readmission (RR=1.10, CI95% 1.07–1.11, p < 0.0001), ICU admission (RR=1.11, CI95% 1.08–1.15, p < 0.0001), acute thromboembolic events (RR=1.14, CI95% 1.07–1.2, p < 0.001), and deep venous thrombosis (RR=1.21, CI95% 1.12–1.32, p < 0.00001). There was no difference in length of hospitalization.

**Conclusion:**

Obesity is a modifiable risk factor that negatively affects COVID-19 outcomes in the older population. Given the prevalence of obesity in our population, primary and secondary obesity prevention is more important than ever.

